# An Intracranial Electroencephalography (iEEG) Brain Function Mapping Tool with an Application to Epilepsy Surgery Evaluation

**DOI:** 10.3389/fninf.2016.00015

**Published:** 2016-04-25

**Authors:** Yinghua Wang, Jiaqing Yan, Jianbin Wen, Tao Yu, Xiaoli Li

**Affiliations:** ^1^State Key Laboratory of Cognitive Neuroscience and Learning and IDG/McGovern Institute for Brain Research, Beijing Normal UniversityBeijing, China; ^2^Center for Collaboration and Innovation in Brain and Learning Sciences, Beijing Normal UniversityBeijing, China; ^3^School of Electrical and Control Engineering, North China University of TechnologyBeijing, China; ^4^Beijing Institute of Functional Neurosurgery, Xuanwu Hospital, Capital Medical UniversityBeijing, China

**Keywords:** intracranial EEG, electrode registration, epilepsy, function mapping, data visualization

## Abstract

**Objects**: Before epilepsy surgeries, intracranial electroencephalography (iEEG) is often employed in function mapping and epileptogenic foci localization. Although the implanted electrodes provide crucial information for epileptogenic zone resection, a convenient clinical tool for electrode position registration and Brain Function Mapping (BFM) visualization is still lacking. In this study, we developed a BFM Tool, which facilitates electrode position registration and BFM visualization, with an application to epilepsy surgeries.

**Methods**: The BFM Tool mainly utilizes electrode location registration and function mapping based on pre-defined brain models from other software. In addition, the electrode node and mapping properties, such as the node size/color, edge color/thickness, mapping method, can be adjusted easily using the setting panel. Moreover, users may manually import/export location and connectivity data to generate figures for further application. The role of this software is demonstrated by a clinical study of language area localization.

**Results**: The BFM Tool helps clinical doctors and researchers visualize implanted electrodes and brain functions in an easy, quick and flexible manner.

**Conclusions**: Our tool provides convenient electrode registration, easy brain function visualization, and has good performance. It is clinical-oriented and is easy to deploy and use. The BFM tool is suitable for epilepsy and other clinical iEEG applications.

## Introduction

Intracranial electroencephalography (iEEG), including electrocorticography (ECoG) or stereo-electroencephalography (sEEG) is the neuroelectrophysiologic signal obtained from implanted subdural or deep electrodes. iEEG has been widely applied in epilepsy surgeries for pre-operative evaluation and functional cortex mapping. Implanted over both pathologic and functionally normal cortex, ECoG/sEEG not only help guide surgical intervention of intractable epilepsy (Wilke et al., 2011), but also provide high spatial-temporal resolution data from affected brain regions (Chang et al., 2010). iEEG is becoming an insight to understand the brain activities. The co-registration of electrodes and the brain structure images, such as those obtained from structural magnetic resonance imaging (MRI), is still a hard problem during application. In neurosurgery departments, the electrode distribution is usually marked by taking some photos during the surgery. The two dimensional image lacks essential coordinate information of each electrode. Therefore, the correct explanation of the electrode positions highly relies on the experience of clinical doctors. There exist several computer-aided electrode registration methods, e.g., using x-ray or high-resolution Computed Tomography (CT) of implanted electrodes to match original MRI images (Miller et al., 2007; Hermes et al., 2010; Dykstra et al., 2012; Wang et al., 2013; Kubanek and Schalk, 2015). However, the brain may have small deformation during electrode implantation, causing error in matching. Therefore, the current electrode registration methods are not perfect. On the other hand, recent studies measuring the network topology and connectivity in patient reveal the roles of cortical networks in epilepsy generation (Franaszczuk et al., 1994; Franaszczuk and Bergey, 1998; Baccalá et al., 2004; Ortega et al., 2008), where localizing the network nodes (i.e., electrode position) is important. The co-registration of electrodes and brain models is limiting the application of epileptogenic network in clinics. Therefore, an easy-to-use, convenient Brain Function Mapping (BFM) tool combining electrode registration and function mapping visualization in clinics is needed indeed.

There have been a few tools for BFM with/without electrode co-registration for academic research. Caret^1^ is a toolbox for structural and functional analyses of the brain cortex. However, this tool lacks functions like electrode registration and graph network visualizations (Xia et al., 2013). The Connectome Viewer is a python-based toolkit to manage, analyze, and visualize connectomes (Gerhard et al., 2011), which focuses on visualization and analysis of multi-modal connection data. Although it has a flexible plugin architecture allowing the development of extensions for extra functionality, electrode registration is still a difficult issue for clinical doctors without a programming background. BioImage Suite 3 is an integrated image analysis software for neuro/cardiac and abdominal image analysis (Papademetris et al., 2006). It ships with a module for editing intracranial EEG electrodes. However, the editor interface is not intuitive. User has to switch between editor/viewer to place electrodes. The learning curve is an obstacle for neurosurgeons. The BrainNet Viewer is a network visualization tool for human brain connectomes, which provides graph-based network visualization with flexible drawing options at user’s request (Xia et al., 2013). However, this tool is developed under Matlab (The MathWorks, Inc., USA), which is a heavyweight, commercial software. This makes it hard to be deployed in clinics. In another word, these research-oriented tools are not very suitable for clinical application.

A suitable electrode registration and function mapping software for clinical environment should meet these criteria:

1. Easy to deploy. The software should be light weight and has as few dependencies as possible. The installation should be as simple as possible. A portable version is preferred. Extra set-ups or installation of dependencies should be avoided as much as possible.
2. Easy to learn and use. The software should be easy-to-use for clinical doctors without technical background. Preferably, the software could be operated simply by mouse clicks and drags.
3. Flexible operations. The software should provide flexible options to meet the user needs. For example, it should provide options to load various neuroimaging data, change the appearance of electrode properties, and adjust the visualization of mappings.

Having these criteria in mind, we have developed a software for epilepsy surgeries, which mainly combines electrode reregistration and BFM. It is a portable and lightweight software that has few dependencies. The software is a single window application, whose controls are easy to understand and manipulate. Most operations can be done with mouse click and drags. It also has flexible options to load various neuroimaging data as well as to control the visualization. Moreover, the results can be saved for further usage and exchange.

## Materials and Methods

### Developing Environment

The BFM Tool was developed in the C# programming language, under a 64 bit Windows 8.1 operating system and Visual Studio 2010 (Microsoft Corporation, Redmond, WA, USA). The software was built upon the Visualization Toolkit (VTK). The VTK is an open-source, freely available software system for three-dimensional (3D) computer graphics, image processing and visualization. VTK supports a wide variety of visualization algorithms including: scalar, vector, tensor, texture, and volumetric methods; and advanced modeling techniques such as: implicit modeling, polygon reduction, mesh smoothing, cutting, contouring, and Delaunay triangulation. More information about the VTK could be obtained in http://www.vtk.org/.

### Workflow

**Figure 1:**
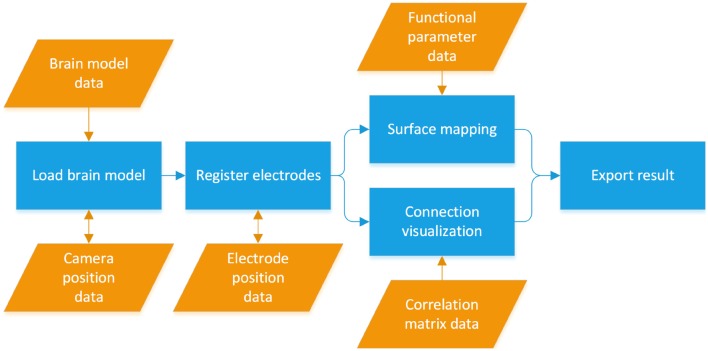
**The workflow of the Brain Function Mapping (BFM) Tool**.

The BFM Tool has four major functions: 3D brain presentation, intracranial electrode registration, mapping of brain function and visualization of brain functional connectivity. The workflow of the software is illustrated in Figure 1. The brain model is loaded from the first model data file. Then, the electrode coordinates are registered, manually or by importing existing location file. Depending on the study, the functional parameters can be mapped either using surface mapping or ball-and-stick connections. Finally, the visualization results can be exported for further use.

### Core Algorithms

Our software does not address brain surface extraction. The 3D brain model requires the model data of vertices and triangle surfaces generated with MRI reconstruction, for example using Statistical Parametric Mapping (SPM) toolbox^2^ on Matlab. The data are converted using a helper tool and loaded at the launch of the software. For electrode placement, the BFM Tool provides an easy and precise method. The electrodes are manually positioned on the brain model space using mouse cursor according to surgery pictures or description of them. On mouse clicking, the two-dimensional coordinates of the mouse cursor are translated into the 3D coordinate of the point on the brain model which the mouse points to. This is done by projecting a 3D ray from the mouse through the camera into the scene, and then check where the ray intersects with the brain surface. This technique is called “ray casting”. Then, their position can be fine-tuned with corresponding controls of the software. The manually placed electrodes are registered using the same coordinates (e.g., the Talairach coordinates or the Montreal Neurological Institute and Hospital (MNI) coordinate) as the 3D brain model. The BFM Tool also has a few plugins to import the electrode coordinates obtained using other approaches, such as: coordinates obtained from stereotactic devices (e.g., ROSA); coordinates obtained from identified electrodes from reconstructed CT images which are scanned after electrode implantation and registered with MRI images; coordinates obtained from X-ray scans. The detail of electrode placement is explained in the “Results” section. For functional mapping and connectivity visualization, the corresponding functional parameters or correlation matrix are calculated using various algorithms, such as ECoG/sEEG voltage, entropy measures, source localization, mutual information, phase synchronization, and so on. Results of other algorithms can be easily imported. Functional parameters can be rendered as balls or maps. For ball plotting, the color and size of each ball represents the parameter’s value of the corresponding electrode; for map plotting, Gaussian interpolation or linear interpolation is used. Functional connectivity is rendered using the ball-and-stick model. The color and thickness of “sticks” can be specified according to correlation strength. See “Results” section for examples of different plots.

### File Formats

We defined several file formats for the BFM Tool, including the brain model file, electrode position file, camera position file, functional parameter file, and correlation matrix file. All files stores values in ASCII format except for the model file. The descriptions of each file type are listed in Table 1. The software comes with detailed specifications of each file type for researchers to implement their own filetype converter plugins easily.

**Table 1:** **File type defined in the BFM Tool**.

Filetype	Description
Brain model file (*.mof)	Brain surface data converted from vertices and triangle faces from other models, e.g., SPM. A helper tool is provided to import the model and change the fineness of the brain surface.
Electrode position file (*.epf)	Contains manually registered electrode coordinates, or coordinates imported from other electrode location files. Users can also edit the coordinates directly in the file.
Camera position file (*.cpf)	Stores the camera view of the brain model, i.e., its distance and rotation angle.
Functional parameter (*.txt)	Contains computed functional parameters. *m × n* vector of parameter values, where *n* is the number of channels. Each value corresponds to a channel, separated by Tab. The file may contain multiple lines (*m* > 1) for a set of functions parameters, e.g., different time stages or indexes.
Correlation Matrix (*.txt)	*n × n* matrix of correlation measure, separated by Tab, where *n* is the number of channels. Value at row *i*, column *j* is the correlation value between channel *i* and *j*.

### Software Download and Installation

The BFM Tool runs under a variety of Windows systems with .NET Framework 4.0 (or above) installed. The software has been successfully tested under Windows 7 and 8, in both 32 bit and 64 bit versions, Windows 7 and above. Earlier Windows versions like Windows XP have not been fully tested. The software package can be downloaded at https://github.com/redyjq/BrainFunctionMapping as an archive of ~90 MB. Follow the instructions inside the downloaded package to install the software.

## Results

### Overview

**Figure 2:**
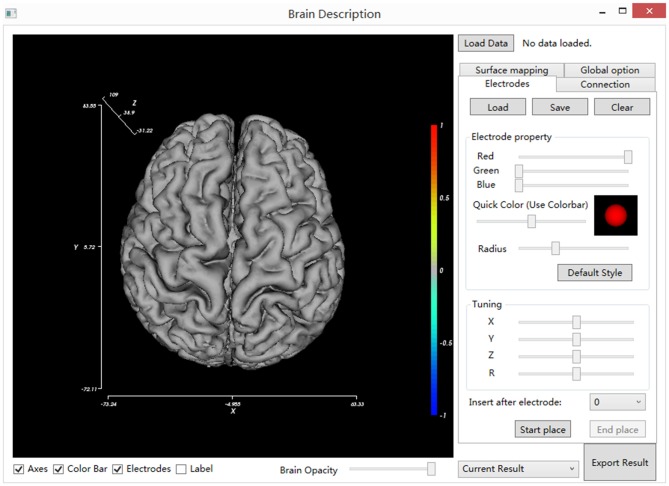
**The main window of BFM Tool.** The main window consists of the main view (left), setting tabs (right), view controls (bottom, under the main view) and general control widgets (top and bottom at right).

Locate and double click on the BrainDescript.exe to launch the BFM Tool. The main window of the BFM Tool is shown in Figure 2. The software has been designed to be intuitive and easy-to-use, so that clinical doctors can easily operate. The graphical user interface consists of two main parts: brain view and controls. The brain view displays the brain model, electrodes, visualized functional parameters, and functional connectivity. User can drag the canvas to change the viewport, or use mouse wheel to zoom the brain model. The controls provide ways to manipulate the display, place/modify electrodes, plot connections and mappings, load/save data, and export results.

#### Brain Model View Controls

**Figure 3:**
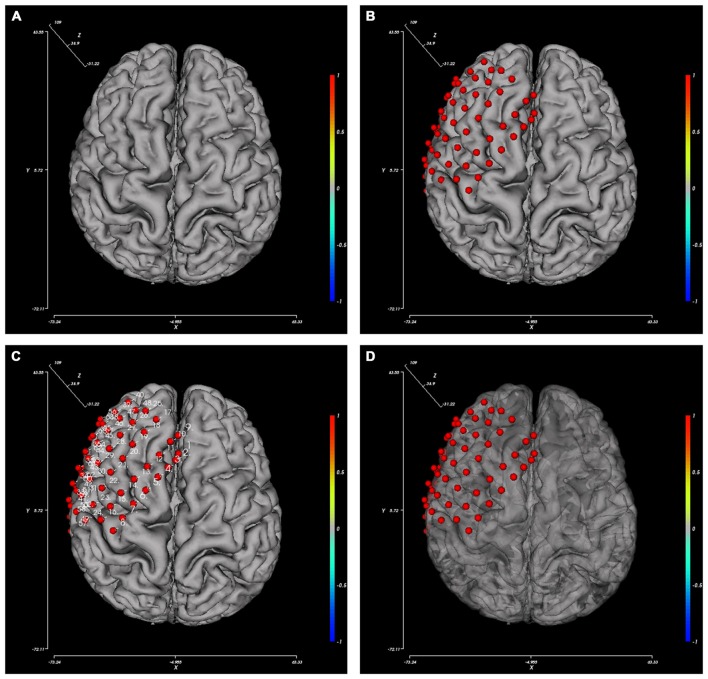
**Pictures generated from different view option combinations. (A)** Brain; **(B)** brain with electrodes; **(C)** brain with electrode and their labels; **(D)** brain with electrodes, the opacity of the brain changed.

The BFM Tool provides several easy and flexible controls over the visualization of brain model and electrodes. The display of these components can be toggled using checkboxes: axes, color bar, electrodes, and electrode labels. To obtain a better view inside the brain surface (e.g., presenting deep electrodes or inter-connections), the brain opacity can be adjusted using the slider below. Figure 3 provides pictures generated from different option combinations.

#### Electrode Operation

**Figure 4:**
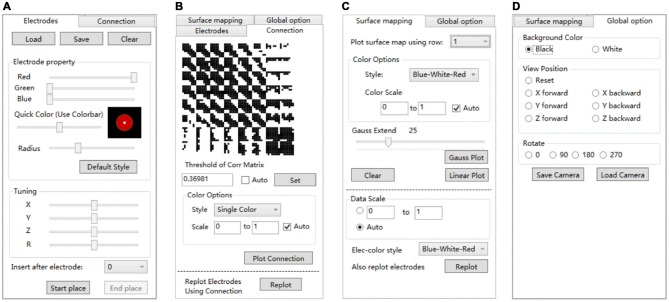
**The setting tabs of BFM Tool. (A)** “Electrodes” tab for electrode registration; **(B)** “Connection” tab for functional connectivity plot; **(C)** “Surface mapping” tab for function mapping plot; **(D)** “Global options” for camera control.

Electrode registration is a major feature of BFM Tool. The software provides an easy way to place electrode in the brain model, with flexible options (Figure 4A). Users can load/save electrode locations or clear the registered electrodes. Before placing an electrode, users can change the color or size of the electrode ball. The color can be adjusted by dragging the “Red”, “Green”, “Blue” sliders or the “Quick Color” slider to choose a color in a predefined color bar. The size is adjusted by dragging the “Radius” slider. The software provides a preview area to check the result. User can reset the electrode ball style and use the “Default Style” button if needed. Then, click “Start place” to start placing electrode. Click on the brain model to place an electrode, and a second click on it to cancel. The coordinates can be adjusted using the “Tuning” sliders. The tuning can be done at a step <1 mm. Therefore, the electrode can have a very high precision. User can also use the “Insert after electrode” combo box to insert after a specified electrode number instead of appending to the electrode list.

#### Connection Operation

**Figure 5:**
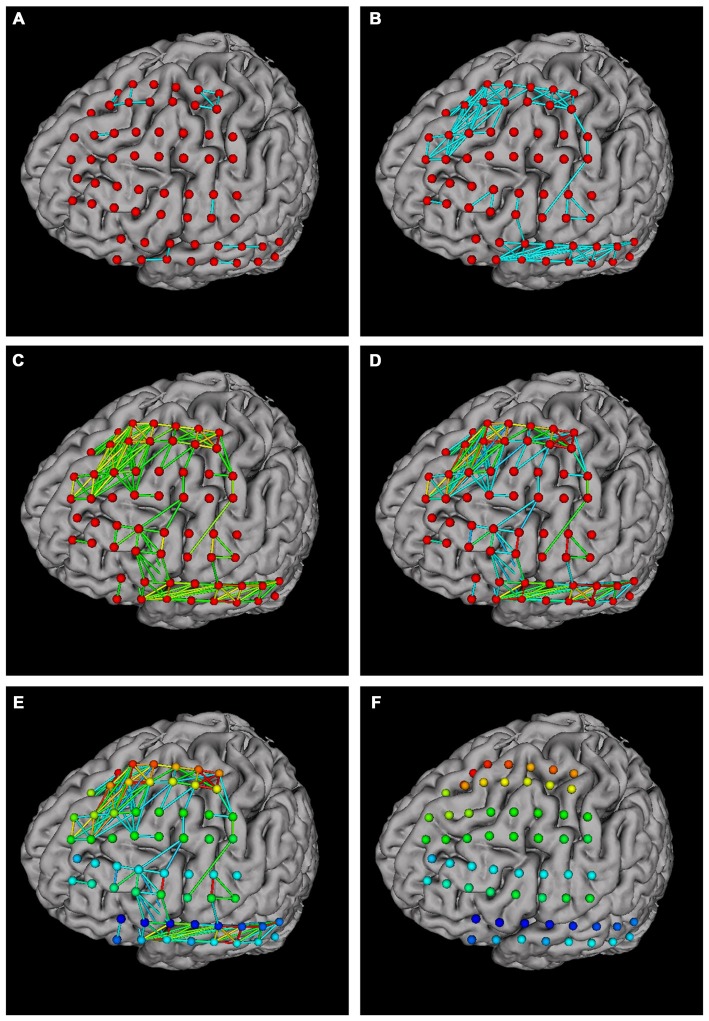
**Ball-and stick plots of functional connections using different plotting options. (A)** Single color sticks with auto connection threshold; **(B)** single color sticks with manual connection threshold, showing more connections; **(C)** color sticks with auto color scale; **(D)** color sticks with manual scale, highlighting strong connections; **(E)** replotting nodes to highlight important nodes; **(F)** hiding connections (setting connection threshold ≥1) for a better view of nodes.

Network analysis is important in brain studies. BFM Tool provides a convenient way to visualize the functional connectivity matrix (network) of multiple brain locations. The “Connection” panel controls the plotting of correlation matrix as ball-and-stick models (Figure 4B). It has a preview of the connection matrix loaded using the “load data” utility. In the preview, black dots represent connections to be plotted. The connection threshold can be determined automatically (with the “auto” checkbox, at a predefined sparsity) or manually (with the input field). Users can choose single color sticks for binary network, or use color sticks for weighted network (with colors representing connection strength). The color scale can also be determined automatically (with the “auto” checkbox), or specified with a range (with the input fields). BFM Tool provides an additional function to replot the electrode nodes according to its connection with other nodes. The color of each node reflects the summed connection strength with other nodes. This makes it easy to check the most important nodes or network hubs. Different ball-and-stick styles are illustrated in Figure 5.

#### Mapping Operation

**Figure 6:**
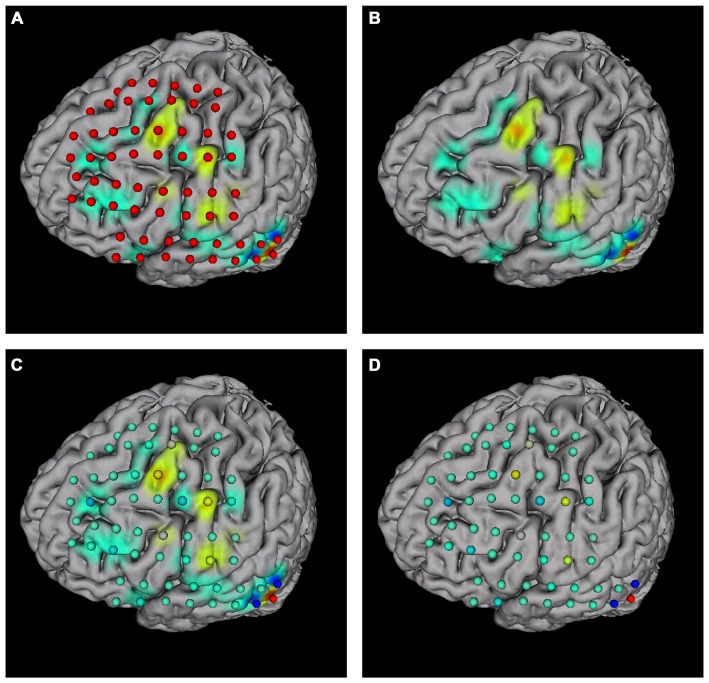
**Surface mapping examples. (A)** Surface map with electrodes; **(B)** surface map without electrodes; **(C)** surface map with replotted nodes; **(D)** replotted nodes without surface map.

The “Surface mapping” tab provides controls of surface map for user defined brain functional parameters (Figure 4C). Here the data is loaded using the “Load data” function, similar to the connection matrix. Users can choose which row of data to use for map plot. The map color transition style and scale can be selected using the controls. BFM Tool provides two map interpolation methods: Linear and Gaussian. The Gaussian interpolation can be adjusted using the “Gauss Extend” slider. The data range to plot can also be selected automatically or manually. Users may also replot the electrodes to highlight important locations. An example of surface mapping is shown in Figure 6.

#### Global Options

BFM Tool provides a few quick options to change the brain view (Figure 4D). The background color can be switched between white and black. A few camera perspectives are provided for quick setting. The camera can be saved for later use.

#### Result Export

The function mapping results can be exported to bmp images for further processing, e.g., preparing for publication. For surface mapping, multiple images are exported for each row of loaded data. This is convenient for investigating the changes of functional parameters over time.

### A Case Study: Language Area Localization

#### Subject

We have applied the BFM Tool in a clinical study of language area localization. The case presented is a patient from Xuanwu Hospital of Capital Medical University in Beijing. The patient was 27 years old, male, and diagnosed with focal epilepsy. Subdural electrodes were implanted to epileptogenic zone localization and eloquent cortex mapping. Before the surgery, we designed a language Event Related Potential (ERP) task for this patient to localize his language cortex. The ECoG signals were recorded using two 32 channel BrainAmp amplifiers (Brain Products GmbH, Germany). Data was sampled at 2500 Hz, and band-passed at 0.03–300 Hz. High resolution T1-weighted MRI scans were obtained for brain surface reconstruction. The protocol of this study has been approved by the Institutional Review Board of Xuanwu Hospital of Capital Medical University, and an informed consent was obtained from the patient.

#### Experimental Design

The experiment paradigm consisted of six production tasks, including picture naming, auditory question response, text question response, sound recognition and naming, word repetition, and word reading. Stimulations were presented on a computer screen and asset of headphones, using the E-Prime software (Psychology Software Tools, Inc., USA). Responses were recorded and synchronized signals were sent to the ECoG recording software. In this article we only present the analysis results for the text question response task to demonstrate the usability of the BFM Tool.

#### Data Processing

Offline data processing and analysis were done with Matlab 2013b software using custom scripts and the EEGLAB toolbox (Delorme and Makeig, 2004). Prior to analysis, data was zero-phase band-pass filtered (0.5–250 Hz). Data was then segmented into epochs of 4300 ms (−800 ms to +3500 ms from stimulation onset). Epochs having apparent noise were dropped. Then ERPs were calculated by averaging the stimulus-aligned epochs. To investigate the functional connectivity between brain areas under the subdural electrodes, we constructed the functional brain networks between the ECoG series by using a phase synchronization method.

#### Visualization

**Figure 7:**
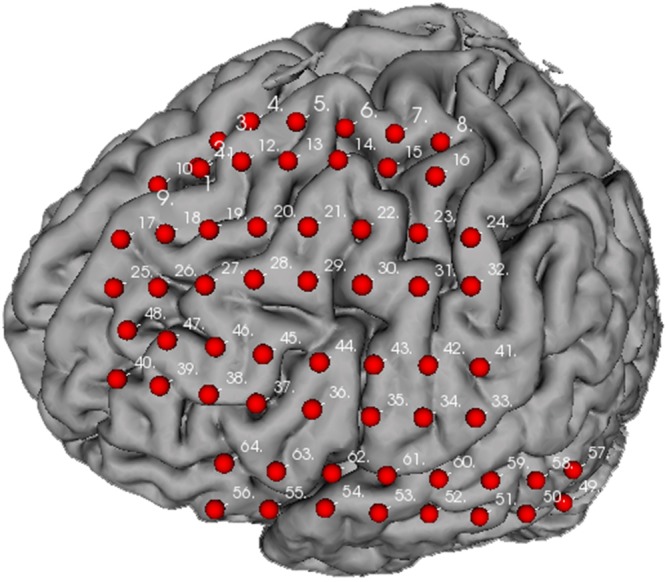
**Electrode distribution for the experimental data**.

Figure 7 illustrates the distribution of implanted electrode arrays on the brain surface. The electrodes were manually registered by a doctor in Xuanwu Hospital according to surgery photo. The brain model used is converted from FMR data using helper plugins provided within the BFM toolbox. The detailed usage of these plugins could be found in the toolbox manual. The electrode arrays were positioned on the frontal gyrus to superior region of precentral gyrus (channel 1–16), frontal gyrus to middle region of postcentral gyrus (channel 17–32), frontal gyrus to inferior part of postcentral gyrus (channel 33–48), and inferior frontal gyrus to posterior middle temporal gyrus (channel 49–64). Manual electrode registration provides an easy and intuitive way for clinical doctors to record and review the electrode implantation scheme.

**Figure 8:**
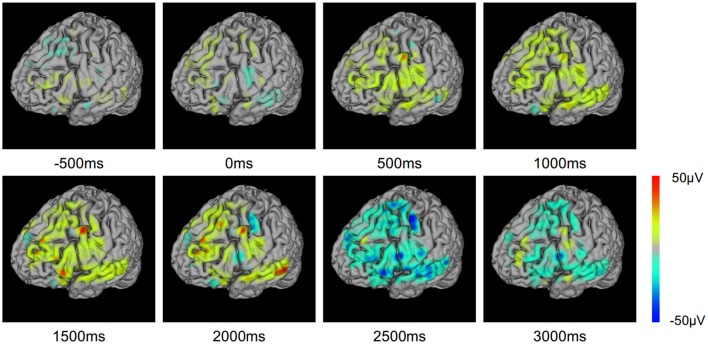
**Event related potential (ERP) voltage map changes through the text question response task**.

To investigate the evolution of ERP on the cortex, we mapped the ERP sequences at different time points on the brain model (Figure 8). The changes in ERP amplitude are clearly revealed. The red areas represent high brain activity at the particular time point. We see that the brain activity increased while the stimulation is presented (0–2500 ms). This indicates the underlying cognitive processing of vocal stimuli. Note that a few sites are more active. These sites may be playing important role in the language processing and understanding. BFM Tool helps clinical doctors to investigate the activity of interesting cortex areas and evaluate their functional roles by designed tasks.

**Figure 9:**
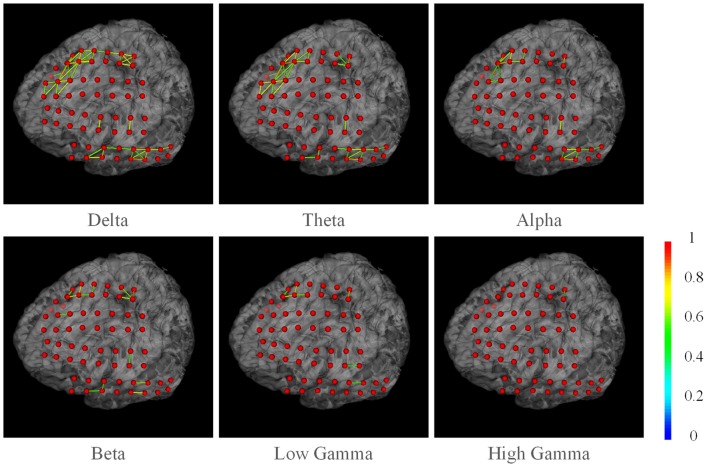
**The phase synchronization network for different frequency bands.** Connections in strength >0.6 are displayed. The color of the sticks represent the connection strength between two nodes.

We also present the functional networks of the electrodes (Figure 9). The functional networks were computed using phase synchronization for Delta (1–4 Hz), Theta (4–8 Hz), Alpha (8–16 Hz), Beta (16–30), Low Gamma (30–80 Hz), and High Gamma (80–250 Hz). The connection sparsity is set to over a threshold of 0.6 in strength of all connections. The stick color represents the synchronization strength between two nodes. The figure shows that for these frequency bands, dominating functional connections are short connections. Two major clusters could be identified: the one spanned frontal gyrus to superior precentral gyrus and the one spanned middle gyrus. This may indicate a possible coordination of the underlying cortex in signal processing. Difference between channels may also provide useful information in clinical studies. For example, the higher synchronization in Gamma band may be a signature of selective attention (Ray et al., 2008). BFM Tool enables researchers to explore more cognitive procedures and functional relationships between different brain areas.

**Figure 10:**
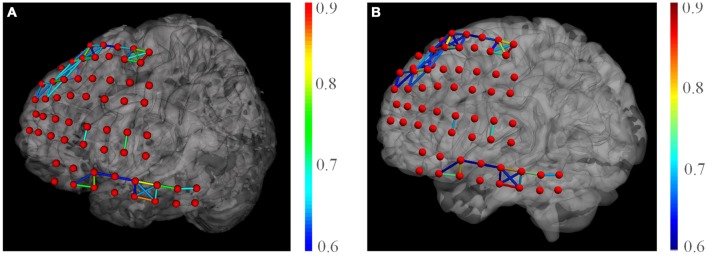
**Comparison of network rendering results by the BFM Tool (A) and the BrainNet Viewer (B)**.

There exist a few toolboxes that could render brain functional networks, e.g., the very popular BrainNet Viewer (Xia et al., 2013). As a comparison, we produced the same network using the BrainNet Viewer and Matlab (see Figure 10). We used the brain model “BrainMesh_Ch2withCerebellum.nv” shipped within the toolbox. Because it only supports specific node file (electrode positions), we manually converted our electrodes into their “.node” format. We tried to use similar settings for node size, edge thickness, network threshold etc. for two toolboxes. The toolbox yielded similar network rendering with our tools. However, as the toolbox is based on Matlab, its rendering is noticeably slower than our tool, especially when loading network matrix and dragging the canvas.

## Discussion

We developed the BFM tool as an assistant software for epilepsy surgeries. The software utilizes functions for brain model loading, electrode registration, functional parameter and connectivity visualization. The software has been evaluated and used in Xuanwu Hospital and received positive feedbacks. We further demonstrated its usability by presenting its application in a clinical study of language area localization.

Our tool provides manual electrode registration that can achieve desirable precision. Some may argue that manual registration relying solely on intraoperative photographs means that only electrodes directly visible from the craniotomy could be localized; stripes inserted at the edges of the craniotomy can hardly be accounted for. Besides, matching gyri and sulci with the arachnoid membrane in place and the electrode grid overlaid can be challenging. Answer to these questions is that the electrode registration for pre-surgery evaluation is a highly specific application. The neurosurgeons do not require to match each electrode to specific gyri and sulci before surgery, but rather which channel shows abnormal activities. Whether a channel covers certain functional area is further determined during surgery. However, adjusting these electrodes to precise positions is possible after surgery. Two ways for doing this are: (1) by performing an x-ray scan of the brain at a particular axial direction, and mapping the electrodes to brain model constructed by previous MRI images (Miller et al., 2007; Kubanek and Schalk, 2015); (2) by performing high-resolution CT scan and co-registrating brain model of CT images and previous MRI images (Hermes et al., 2010; Wang et al., 2013). The first method is simpler, and the second one is more precise. For example, Dykstra et al. (2012) used the CT method and found their result a median error of 3 mm. However, even the CT-based method may produce minor error, because the brain tissues may have deformed after the implantation, causing slightly different modeling results by CT and MRI. This kind of deformation can be easily corrected using our software. Gupta et al. (2014) developed a method for localizing ECoG electrodes on the cortical anatomy without post-implantation imaging. But this method requires a clinical neural navigation device (Gupta et al., 2014). On the other hand, our tool aids experienced doctors (often who performed the implantation surgery) to manually register the electrodes. This avoids the drawbacks of existing methods and is more precise.

Unlike developed toolboxes, the BFM tool doesn’t try to integrate the MRI reconstruction, functional parameter algorithms or brain connectome measures. More functions do not necessarily make it a better software for clinical use. Quite the contrary, the increased complexity makes clinical doctors more difficult to learn the software. By providing canonical interfaces, researchers or clinical doctors could apply their preferred tools or measures.

The BFM tool is developed with C# and VTK. This provides the tool with higher performance than those written in scripting language. For example, Matlab programs suffer from high consumption and slow loop execution as well as graphical rendering speed (Xia et al., 2013). The performance of the BFM tool is advantageous in clinical environments.

The BFM tool provides network visualization necessary for brain function area evaluations. However, it is not trying to become a replacement of network visualization toolboxes such as GAT (Hosseini et al., 2012), PANDA (Cui et al., 2013), Connectome Viewer (Gerhard et al., 2011). For example, Connectome Viewer provides functions to integrate multi-modal data and provides connectome analysis, graph metrics, and group-based statistics to better meet research needs. For such more sophisticated applications, researchers could consider a more suitable toolbox. But on the other hand, for network visualization in ECoG/sEEG studies, this tool is indeed a good tool to present results for different brain studies owing to its high rendering performance, easy operation, and flexible interfaces (see Figures 8, 9).

Although the BFM tool provides a convenient assistant tool for pre-operative evaluation, diagnsis of epilepsy still highly relies on clinical doctors’ judgment based on ECoG waveforms, clinical symptoms, MRI images and other measurements. We will keep improving the current version by fixing bugs and adding more functions as required by clinical doctors, such as customizing labels, node group manipulation etc. to make it a better toolbox for clinical use.

In conclusion, our toolbox can be very useful in many research and clinical areas. Besides pre-surgery evaluation of epilepsy seizure foci, it could also be applied in iEEG-based cognitive researches, teaching illustration, etc. Due to its flexible interface, the toolbox is extendable to integrate with or import from well-developed third-party software, e.g., SPM or Matlab, thus making it more powerful for a wider range of users.

## Author Contributions

YW and JY designed and developed the software. YW, JY, and XL wrote the article. JW and Dr. TY provided the experimental data. We thank Dr. TY for testing the early builds of this software and providing valuable suggestions in software requirements and functionality. YW and JY equally contributed to this work.

## Conflict of Interest Statement

The authors declare that the research was conducted in the absence of any commercial or financial relationships that could be construed as a potential conflict of interest.

## Footnotes

^1^http://brainvis.wustl.edu/wiki/index.php/Caret:About

^2^http://www.fil.ion.ucl.ac.uk/spm/
